# Prevalence, antimicrobial susceptibility and virulence gene profiles of *Arcobacter* species isolated from human stool samples, foods of animal origin, ready-to-eat salad mixes and environmental water

**DOI:** 10.1186/s13099-021-00472-y

**Published:** 2021-12-20

**Authors:** Dainius Uljanovas, Greta Gölz, Vanessa Brückner, Audrone Grineviciene, Egle Tamuleviciene, Thomas Alter, Mindaugas Malakauskas

**Affiliations:** 1 Department of Food Safety and Quality, Faculty of Veterinary Medicine, Veterinary Academy, Lithuanian University of Health Sciences, Kaunas, Lithuania; 2 Institute of Food Safety and Food Hygiene, Freie Universität Berlin, Berlin, Germany; 3 Kaunas Clinical Hospital Microbiology Laboratory, Medical Academy, Lithuanian University of Health Sciences, Kaunas, Lithuania; 4 Department of Pediatrics, Medical Academy, Lithuanian University of Health Sciences, Kaunas, Lithuania

**Keywords:** *Arcobacter*, Humans, Food, Environmental water, Prevalence, Antimicrobial susceptibility, Virulence genes

## Abstract

**Background:**

Members of the genus *Arcobacter* are considered as emerging zoonotic food and waterborne pathogens that cause gastroenteritis and bacteremia in humans. However, the potential risk that *Arcobacter* species pose to public health remains unassessed in various countries, including Baltic states. Therefore, the aim of this study was to determine the prevalence, antimicrobial susceptibility and presence of putative virulence genes of *Arcobacter* isolates recovered from humans, food products and environmental water in Lithuania.

**Results:**

A total of 1862 samples were collected and examined from 2018 to 2020 in the city of Kaunas. Overall, 11.2% (n = 208) of the samples were positive for the presence of *Arcobacter* spp. The highest prevalence was detected in chicken meat (36%), followed by environmental water (28.1%), raw cow milk (25%), ready-to-eat salad mixes (7.1%) and human stool (1.7%). *A. butzleri* was the most frequently isolated species (n = 192; 92.3%), followed by *A. cryaerophilus* (n = 16; 7.7%). *Arcobacter* spp. antimicrobial susceptibility testing revealed unimodally distributed aggregated minimal inhibitory concentrations (MICs) for gentamicin, tetracycline, ciprofloxacin, ampicillin and erythromycin. However, a bimodal distribution for azithromycin was found with 96.2% of determined MICs above the epidemiological cut-off value (ECOFF) defined for *Campylobacter jejuni* (0.25 µg/ml). Majority of the *Arcobacter* isolates (n = 187; 89.9%) showed high susceptibility to ciprofloxacin with MICs below or equal to the ECOFF value of 0.5 µg/ml. The putative virulence genes *cadF* (100%), *ciaB* (100%), *cj1349* (99%), *tlyA* (99%), *mviN* (97.9%) and *pldA* (95.8%) were the predominant genes detected among *A. butzleri* isolates. In contrast, the *mviN* and *ciaB* genes were present in all, whereas *cj1349* (12.5%), *tlyA* (25%) and *hecA* (12.5%) were only detected in few *A. cryaerophilus* isolates.

**Conclusions:**

Our results demonstrate that food products and environmental water in Lithuania are frequently contaminated with *Arcobacter* spp. that carry multiple putative virulence genes. Furthermore, *A. butzleri* were isolated from 1.7% of inpatients. Fluoroquinolones and aminoglycosides were found to be more effective against *Arcobacter* in comparison to other antimicrobial agents. However, further studies are needed to determine the pathogenic mechanisms and factors that facilitate the spread of *Arcobacter* infections.

## Background

The genus *Arcobacter* was proposed in 1991 [1] based on DNA–rRNA, DNA–DNA hybridization and immunotyping analysis of *Campylobacter* and related organisms. Since then, a total of 29 species for this genus have been described [2]. Recently, Pérez-Cataluña et al. [3] proposed to divide the genus into seven different genera, however, the newly proposed classification is still under debate [4, 5]. Due to their ability to form biofilms on abiotic surfaces and survive in different conditions, *Arcobacter* species are widely distributed throughout the food chain and environment [6, 7]. *Arcobacter* spp. have been isolated from various sources: farm environment, animals, vegetables and food products of animal origin (at the processing stage and retail), food-processing facilities, environmental waters, sewage and floodwater [8–11]. Consumption of contaminated food of animal origin (meat, milk, seafood), vegetables or water is considered as the main route of transmission to humans [6]. Clinical symptoms associated with *Arcobacter* gastrointestinal infections in humans include persistent aqueous diarrhea, abdominal pain and fever [7, 8]. However, infections of immunocompromised patients can result in bacteremia, peritonitis and endocarditis [6, 12, 13]. The majority of *Arcobacter* infections among humans and animals are caused by *Arcobacter* (*A.*) *butzleri*, *A. cryaerophilus* and, to a lesser extent, *A. skirrowii* and *A. thereius* [14–16]. Given that there are no routine diagnostic procedures designed for the detection of *Arcobacter* spp., their prevalence and significance of infections might be underestimated. To date, the reported prevalence of *Arcobacter* among humans range from 0.3 to 4% [17, 18]. Recent studies have shown that *Arcobacter* was the second and fourth most common bacterial pathogen isolated from human stool samples in Germany and Belgium, respectively [14, 19].

Similar to *Campylobacter*, *Arcobacter* cause self-limiting infections which do not require antimicrobial therapy, although cases of severe and chronic enteritis may necessitate the use of antibiotics [8]. Fluoroquinolones, tetracyclines, macrolides, aminoglycosides and a combination of β-lactam antibiotics with β-lactamase inhibitors are suggested as viable treatment options in these cases [11, 20]. Nonetheless, a recent meta-analysis indicated that between 69.3 and 99.2%, 4.3–14%, 10.7–39.8% and 0.8–7.1% of *Arcobacter* spp. isolates have shown reduced susceptibility to penicillins, fluoroquinolones, macrolides and tetracyclines, respectively [21]. Furthermore, other studies revealed reduced susceptibility to multiple antimicrobials in up to 89% of *Arcobacter* strains isolated from human clinical samples, food products and environment [22–24].

*In vitro* human and animal cell culture assays have shown that *Arcobacter* spp. have pathogenic properties (adhesion, invasion, cytotoxicity and ability to upregulate interleukin-8 expression) that are significant for the colonization of host tissues and establishing infection [25, 26]. Several studies investigated adhesive, invasive and/or cytotoxic capabilities of *A. butzleri* strains isolated from various sources (reviewed by Chieffi et al.) [7]. In summary, 25–100% of tested strains were able to induce cytotoxic effects, 12.5–100% to adhere and 0–100% to invade different cell lines (Caco-2, Hep-2, Vero, HT-29, HeLa). Bücker et al. [27] observed that infection of human colonic cells (HT-29/B6) with *A. butzleri* results in a decreased expression of integral transmembrane proteins (claudin-1, -5, -8) and induction of epithelial apoptosis, which are mechanisms that are consistent with a leak flux type of diarrhea. The analysis of *A. butzleri* RM4018 whole genome sequence revealed the presence of ten putative virulence-associated genes (*cadF*, *cj1349*, *ciaB*, *mviN*, *pldA*, *tlyA*, *irgA*, *hecA*, *hecB*, *iroE*) that have homologs in other pathogens (e.g. *C. jejuni*, *V. cholerae* and uropathogenic *E. coli*) [28].

To date, no studies were carried out to determine the *Arcobacter* prevalence among humans in Lithuania or other Baltic states. The absence of data on contamination of food products and environment, antimicrobial resistance, and occurrence of putative virulence genes complicates the assessment of the potential risk to public health. Therefore, the objectives of this study were (i) to determine the prevalence of *Arcobacter* spp. in different sources (human stool samples, foods of animal origin, ready-to-eat salad mixes and environmental water), (ii) to assess the antimicrobial susceptibility patterns of isolated bacteria and to obtain minimal inhibitory concentration (MIC) distribution data, and (iii) to evaluate the pathogenic potential of strains by determining the occurrence of virulence-associated genes.

## Results

### Prevalence of *Arcobacter*

As summarized in Table 1, *Arcobacter* spp. were isolated from 208 (11.2%) out of the 1862 samples tested. The isolation rate of *Arcobacter* varied among different sample types; the highest prevalence was in chicken meat (36%), followed by environmental water (28.1%), raw cow milk (25%), ready-to-eat (RTE) salad mixes (7.1%) and human stool (1.7%). Only two species were identified by multiplex PCR and *rpoB* sequencing: *A. butzleri* (192 of 208 isolates, 92.3%) and *A. cryaerophilus* (16 of 208 isolates, 7.7%). *A. butzleri* was recovered from all sources, whereas *A. cryaerophilus* was only isolated from RTE salads (5 of 99 samples, 5.1%), surface waters (6 of 128 samples, 4.7%) and chicken meat (5 of 331 samples, 1.5%). *A. butzleri* was the predominant species in most sources except RTE salad mixes, where *A. cryaerophilus* was more prevalent.

**Table 1:** Prevalence of *Arcobacter* spp. in the examined samples

Matrix	Sampling period^a^	No. of samples	No. of positive samples (%)			
			*Arcobacter* spp.	*A. butzleri*	*A. cryaerophilus*	*A. skirrowii*
Chicken meat	10.2018–09.2019	331	119 (36)^a^	114 (95.8)	5 (4.2)	–
Raw cow milk	01.2019–12.2019	104	26 (25)^b^	26 (100)	–	–
RTE salad mixes	11.2018–03.2019 and 05.2019–10.2019	99	7 (7.1)^c^	2 (28.6)	5 (71.4)	–
Environmental water	12.2018–11.2019	128	36 (28.1)^a,b^	30 (83.3)	6 (16.7)	–
Human stool	03.2019–02.2020	1200	20 (1.7)^d^	20 (100)	–	–
Total		1862	208 (11.2)	192 (92.3)^e^	16 (7.7)^f^	–

^a−d^ Values in the same column denoted by different superscript letters are significantly different (P < 0.05)

^e,f^ Values in the same row denoted by different superscript letters are significantly different (P < 0.05)

### Antimicrobial susceptibility

**Fig. 1:**
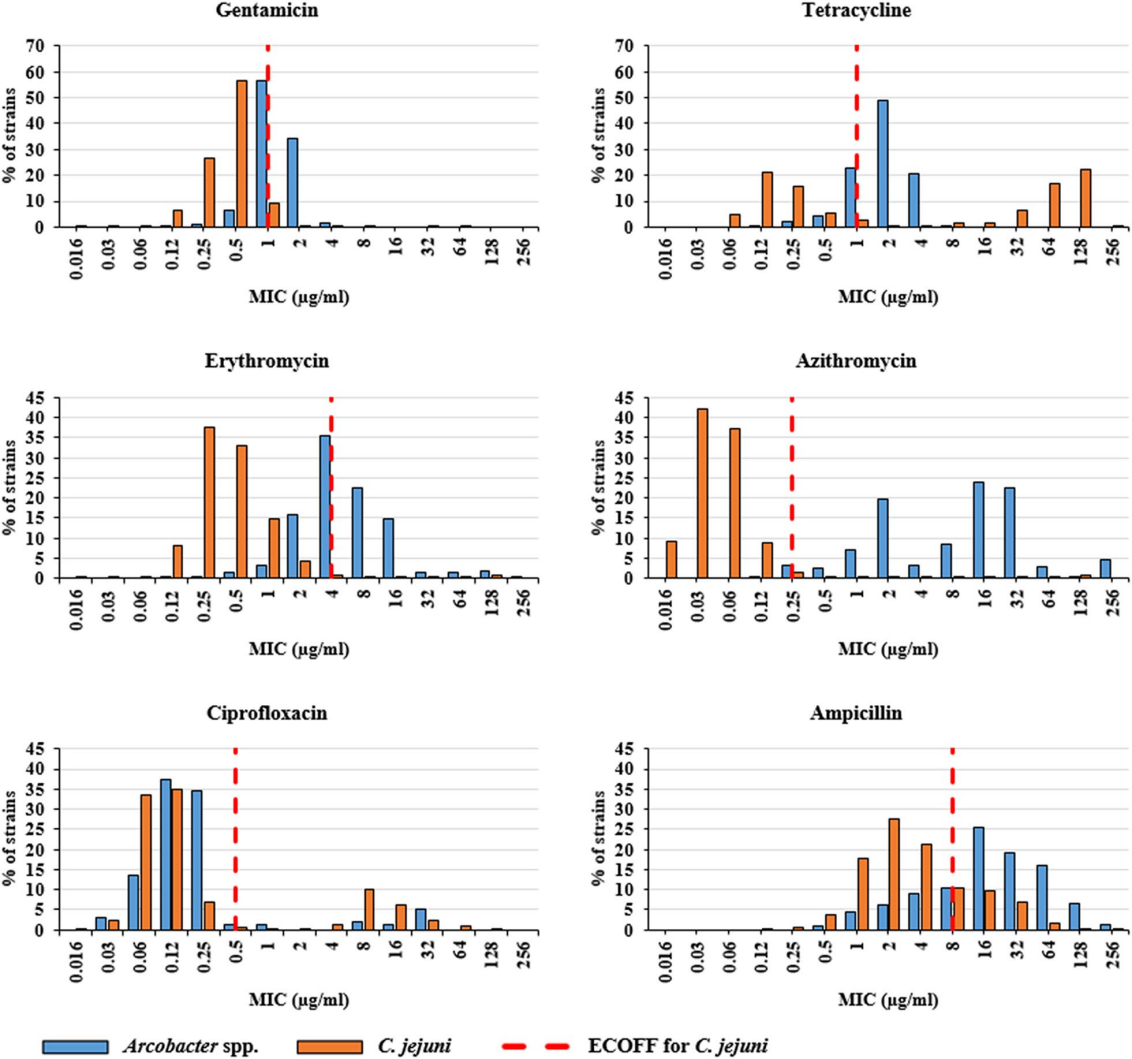
Aggregated MIC distributions of *Arcobacter* spp. isolated from food, environmental water and human stool samples. In addition to the MICs of the *Arcobacter* spp. strains (blue) isolated in our study, the epidemiological cut-off values (ECOFFs) (red broken lines) and MICs of *C. jejuni* (orange) from the EUCAST database are also shown [29]. E-test half-log values were rounded up to the next highest value of the standard doubling dilution scale. In order to prevent numerical dominance of EUCAST MIC distributions, number of isolates at each MIC value are presented as percentage of the total observations from individual dataset

The results of antimicrobial susceptibility testing (AST) of 208 *Arcobacter* spp. isolates revealed unimodally distributed aggregated minimal inhibitory concentrations (MICs) for gentamicin, tetracycline, ciprofloxacin, ampicillin and erythromycin, whereas a bimodal distribution for azithromycin was detected (Fig. 1). The MICs of gentamicin and tetracycline were distributed around the epidemiological cut-off (ECOFF) values defined for *C. jejuni* (1 µg/ml for both antimicrobials), with no interspecies differences (Fig. 1; Table 2). In case of gentamicin, MIC values ranged from 0.125 to 4 µg/ml (mode = 1 µg/ml), while for tetracycline MICs ranging from 0.125 to 8 µg/ml (mode = 2 µg/ml) were observed (Table 2). The range of MICs for macrolides was wider in comparison to other tested antimicrobial agents. For erythromycin, MIC values were distributed around the ECOFF for *C. jejuni* and peaked at 4 µg/ml (Fig. 1). However, 96.2% of determined MICs (ranging from 0.5 to > 256 µg/ml; Table 2) for azithromycin were above the ECOFF of *C. jejuni* (0.25 µg/ml) with peaks at 2 µg/ml and 16 µg/ml (Fig. 1). Additionally, 67.7% (130/192) of *A. butzleri* and 12.5% (2/16) of *A. cryaerophilus* isolates formed a subpopulation (MICs ≥ 8 µg/ml; Fig. 1), which displayed reduced susceptibility to azithromycin. Most isolates (190/208, 91.3%) were highly susceptible to ciprofloxacin with MICs distributed on the lower end of the tested concentration range (from 0.032 to 1 µg/ml, mode = 0.125 µg/ml; Table 2). However, 8.9% (17/192) of *A. butzleri* and 6.3% (1/16) of *A. cryaerophilus* strains showed elevated MICs for ciprofloxacin (≥ 8 µg/ml; Table 2). The MICs of ampicillin (ranging from 0.5 to > 256 µg/ml, mode = 16 µg/ml; Table 2) were distributed around the ECOFF for *C. jejuni* (8 µg/ml; Fig. 1). The majority of *A. cryaerophilus* isolates (13/16, 81.3%) displayed MIC values that were below or equal to the ECOFF, while MICs of 72.9% (170/192) *A. butzleri* isolates were above and 2–32 times higher (Table 2).

**Table 2:** MIC data on six antimicrobial agents for 192 *A. butzleri* and 16 *A. cryaerophilus* isolates

ATM^a^	Species	Source	No. of isolates with MIC (µg/ml) of:															
			0.03	0.06	0.13	0.25	0.5	1	2	4	8	16	32	>32	64	128	256	>256
AM	*A. butzleri*	Chicken meat					2	8	8	11	11	27	26		15	6		
	*A. cryaerophilus*									2	3							
	*A. butzleri*	Environmental water						1	1	1		4	5		8	7	1	2
	*A. cryaerophilus*								1	2	2	1						
	*A. butzleri*	RTE salad mixes													2			
	*A. cryaerophilus*								1		2	1	1					
	*A. butzleri*	Raw cow milk							1	1		11	6		7			
		Human stool							1	2	4	9	2		1	1		
AZ	*A. butzleri*	Chicken meat			1	4	1	6	18	5	11	30	26		2		6	4
	*A. cryaerophilus*						1	2	1	1								
	*A. butzleri*	Environmental water						1	1		4	11	10		3			
	*A. cryaerophilus*					1	1	2	1		1							
	*A. butzleri*	RTE salad mixes											2					
	*A. cryaerophilus*					2	2					1						
	*A. butzleri*	Raw cow milk						3	12	1	1	2	6		1			
		Human stool						1	8	0	1	6	3			1		
GM	*A. butzleri*	Chicken meat					6	67	41									
	*A. cryaerophilus*					1	1	1	2									
	*A. butzleri*	Environmental water					2	22	6									
	*A. cryaerophilus*					1	1	1	2	1								
	*A. butzleri*	RTE salad mixes						2										
	*A. cryaerophilus*				1				4									
	*A. butzleri*	Raw cow milk						13	11	2								
		Human stool					3	12	5									
TC	*A. butzleri*	Chicken meat				1	4	31	56	22								
	*A. cryaerophilus*					2			3									
	*A. butzleri*	Environmental water					2		14	13	1							
	*A. cryaerophilus*					1	3		2									
	*A. butzleri*	RTE salad mixes								2								
	*A. cryaerophilus*				1			3	1									
	*A. butzleri*	Raw cow milk						7	17	2								
		Human stool						7	9	4								
EM	*A. butzleri*	Chicken meat			1	1	1	2	14	43	30	12	3		2	4		1
	*A. cryaerophilus*						1	1	1	2								
	*A. butzleri*	Environmental water							4	6	11	8			1			
	*A. cryaerophilus*						1	1	2	2								
	*A. butzleri*	RTE salad mixes									1	1						
	*A. cryaerophilus*							2	2			1						
	*A. butzleri*	Raw cow milk						1	6	10	3	6						
		Human stool							4	11	2	3						
CI	*A. butzleri*	Chicken meat	1	8	49	35	3	2			4	3		9				
	*A. cryaerophilus*		2			3												
	*A. butzleri*	Environmental water		4	11	14		1										
	*A. cryaerophilus*				1	4								1				
	*A. butzleri*	RTE salad mixes				2												
	*A. cryaerophilus*		1		3	1												
	*A. butzleri*	Raw cow milk	1	10	7	7								1				
		Human stool	1	6	7	6												

^a^ ATM = antimicrobials: AM = ampicillin, AZ = azithromycin, GM = gentamicin, TC = tetracycline, EM = erythromycin, CI = ciprofloxacin

### Occurrence of putative virulence genes

The detection of ten putative virulence genes by PCR in 192 *A. butzleri* and 16 *A. cryaerophilus* isolates from food, environmental water and human clinical samples is summarized in Table 3. Regardless of species, the *ciaB* gene was present in all *Arcobacter* isolates. Similarly, 98.1% of tested strains harbored the *mviN* gene, while other genes were less frequently detected. Overall, all putative virulence-associated genes were detected among the analyzed *A. butzleri* isolates, whereas only five were identified in *A. cryaerophilus*. The majority of *A. butzleri* isolates carried *cadF* (100%), *ciaB* (100%), *cj1349* (99%), *tlyA* (99%), *mviN* (97.9%) and *pldA* (95.8%) genes. However, lower detection rates were observed for *hecB* (38.5%), *hecA* (20.3%), *iroE* (18.8%) and *irgA* (12.5%). In contrast, for *A. cryaerophilus* only the *mviN* and *ciaB* genes were detected in all isolates, and *tlyA* was found only in four (25%) isolates. Furthermore, the *cj1349* and *hecA* genes were detected in two (12.5%) *A. cryaerophilus* isolates.

**Table 3:** Distribution of putative virulence genes in 208 *Arcobacter* isolates from various sources

Species	Source (n)	No. of isolates generating a virulence-associated gene amplicon (%)									
		*cadF*	*cj1349*	*mviN*	*ciaB*	*pldA*	*tlyA*	*hecA*	*hecB*	*iroE*	*irgA*
*A. butzleri*	Human stool (20)	20 (100)	20 (100)	20 (100)	20 (100)	19 (95)	20 (100)	1 (5)	12 (60)	0 (0)	0 (0)
	Food (142)	142 (100)	140 (98.6)	138 (97.2)	142 (100)	135 (95.1)	140 (98.6)	26 (18.3)	42 (29.6)	20 (14.1)	11 (7.7)
	Chicken meat (114)	114 (100)	112 (98.2)	112 (98.2)	114 (100)	109 (95.6)	113 (99.1)	24 (21.1)	29 (25.4)	20 (17.5)	11 (9.6)
	Raw cow milk (26)	26 (100)	26 (100)	24 (92.3)	26 (100)	24 (92.3)	25 (96.2)	0 (0)	11 (42.3)	0 (0)	0 (0)
	RTE salad mixes (2)	2 (100)	2 (100)	2 (100)	2 (100)	2 (100)	2 (100)	2 (100)	2 (100)	0 (0)	0 (0)
	Environmental water (30)	30 (100)	30 (100)	30 (100)	30 (100)	30 (100)	30 (100)	12 (40)	20 (66.7)	16 (53.3)	13 (43.3)
	Total (192)	192 (100)	190 (99)	188 (97.9)	192 (100)	184 (95.8)	190 (99)	39 (20.3)	74 (38.5)	36 (18.8)	24 (12.5)
*A. cryaerophilus*	Food (10)	0 (0)	1 (10)	10 (100)	10 (100)	0 (0)	1 (10)	2 (20)	0 (0)	0 (0)	0 (0)
	Chicken meat (5)	0 (0)	1 (20)	5 (100)	5 (100)	0 (0)	1 (20)	1 (20)	0 (0)	0 (0)	0 (0)
	RTE salad mixes (5)	0 (0)	0 (0)	5 (100)	5 (100)	0 (0)	0 (0)	1 (20)	0 (0)	0 (0)	0 (0)
	Environmental water (6)	0 (0)	1 (16.7)	6 (100)	6 (100)	0 (0)	3 (50)	0 (0)	0 (0)	0 (0)	0 (0)
	Total (16)	0 (0)	2 (12.5)	16 (100)	16 (100)	0 (0)	4 (25)	2 (12.5)	0 (0)	0 (0)	0 (0)

Overall, 7.3% (14/192) of *A. butzleri* isolates (seven from chicken meat and seven from environmental water) harbored all ten putative virulence genes (Table 4). Meanwhile, for *A. cryaerophilus*, a maximum of four genes was detected in two isolates (one from meat and one from water) by our PCR; however, the majority of isolates (10/16, 62.5%) simultaneously carried two genes (*ciaB* and *mviN*). Among *A. butzleri* isolates, the most common (96/192, 50%) virulence gene pattern was *ciaB*, *mviN*, *pldA*, *tlyA*, *cj1349* and *cadF*. This profile was observed in isolates from chicken meat, raw cow milk, environmental water and human stool samples with rates of 60.5%, 50%, 23.3%, and 35%, respectively. Only 35.2% of *A. butzleri* strains from food samples carried at least seven putative virulence genes, while higher rates were determined for strains from human stool samples (60%) and environmental water (76.7%).

**Table 4:** Virulence gene profiles of *A. butzleri* and *A. cryaerophilus* isolates

Virulence patterns	No. of *A. butzleri* isolates (%)					No. of *A. cryaerophilus* isolates (%)		
	Chicken meat (n = 114)	Raw milk (n = 26)	Salad mixes (n = 2)	Water (n = 30)	Human stool (n = 20)	Chicken meat (n = 5)	Salad mixes (n = 5)	Water (n = 6)
2 genes								
* ciaB, mviN*						3 (60)	4 (80)	3 (50)
3 genes								
* ciaB, mviN, tlyA*						1 (20)		2 (33.3)
* ciaB, mviN, hecA*							1 (20)	
4 genes								
* ciaB, mviN, hecA, cj1349*						1 (20)		
* ciaB, mviN, tlyA, cj1349*								1 (16.7)
5 genes								
* ciaB, pldA, tlyA, cj1349, cadF*	1 (0.9)	1 (3.8)						
* ciaB, mviN, tlyA, cj1349, cadF*	1 (0.9)	1 (3.8)						
* ciaB, mviN, pldA, tlyA, cadF*	1 (0.9)							
6 genes								
* ciaB, mviN, pldA, tlyA, cj1349, cadF*	69 (60.5)	13 (50)		7 (23.3)	7 (35)			
* ciaB, mviN, tlyA, hecB, cj1349, cadF*	1 (0.9)	1 (3.8)			1 (5)			
* ciaB, mviN, tlyA, cj1349, iroE, cadF*	1 (0.9)							
* ciaB, pldA, tlyA, hecB, cj1349, cadF*		1 (3.8)						
* ciaB, mviN, pldA, hecB, cj1349, cadF*		1 (3.8)						
7 genes								
* ciaB, mviN, pldA, tlyA, hecA, cj1349, cadF*					1 (5)			
* ciaB, mviN, pldA, tlyA, hecB, cj1349, cadF*	3 (2.6)	8 (30.8)		2 (6.7)	11 (55)			
* ciaB, mviN, pldA, tlyA, cj1349, iroE, cadF*	10 (8.8)			3 (10)				
* ciaB, mviN, pldA, tlyA, irgA, cj1349, cadF*	1 (0.9)							
* ciaB, mviN, pldA, hecA, hecB, cj1349, cadF*	1 (0.9)							
* ciaB, mviN, pldA, tlyA, hecA, hecB, cadF*	1 (0.9)							
8 genes								
* ciaB, mviN, pldA, tlyA, hecA, hecB, cj1349, cadF*	14 (12.3)		2 (100)	5 (16.7)				
* ciaB, mviN, pldA, tlyA, irgA, cj1349, iroE, cadF*	1 (0.9)							
9 genes								
* ciaB, mviN, pldA, tlyA, irgA, hecB, cj1349, iroE, cadF*	1 (0.9)			6 (20)				
* ciaB, mviN, tlyA, irgA, hecA, hecB, cj1349, iroE, cadF*	1 (0.9)							
10 genes								
* ciaB, mviN, pldA, tlyA, irgA, hecA, hecB, cj1349, iroE, cadF*	7 (6.1)			7 (23.3)				

Statistical analysis revealed no significant differences between the occurrence of *ciaB*, *mviN*, *pldA*, *tlyA*, *cj1349*, *cadF* genes in *A. butzleri* isolates of different origin (food, environmental water and human stool samples, P > 0.05). None of the tested *A. butzleri* isolates from human stool specimens carried the *iroE* and *irgA* genes, while *hecA* was detected in one isolate (5%). The highest detection rates of *hecA* (40%), *irgA* (43.3%) and *iroE* (53.3%) were observed among isolates from environmental water. These rates were significantly higher (P < 0.05) than the ones determined for isolates from food (18.3%, 7.7% and 14.1%, respectively). Furthermore, among isolates from human stool and environmental water, the occurrence of *hecB* gene (60% and 66.7%) was significantly higher (P < 0.05) in comparison with isolates from food samples (29.6%). The *cadF* and *pldA* genes were not detected in *A. cryaerophilus* isolates from meat, RTE salad mixes, and water, while these genes were present in majority of *A. butzleri* isolates (rates ranging between 95.6 and 100%) of same origin.

## Discussion

### *Arcobacter* spp. prevalence in various types of samples

Research progress on prevalence and pathogenicity has led *A. butzleri* and *A. cryaerophilus* to be ranked as serious hazards to human health by the International Commission on Microbiological Specifications for Foods (ICMSF, 2002) [30]. However, due to missing standardized isolation and identification methods, *Arcobacter* spp. prevalence data in various countries remain undetermined. This appears to be the first study of its kind analyzing the prevalence of *Arcobacter* spp. in different sources in Lithuania by using *Arcobacter*-specific detection methods combined with molecular confirmation.

Within the present study, *Arcobacter* spp. were isolated from 20 out of a total of 1200 (1.7%) human stool samples tested. There are no epidemiological data provided by other Baltic countries that could be used for comparative analysis. However, this finding is consistent with studies from Belgium and Portugal, where *Arcobacter* spp. were detected in 1.3% (89/6774) and 1.7% (5/298) of clinical stool samples respectively [14, 31]. Other studies, conducted in Turkey, Germany, India, Chile and Belgium, reported different prevalence rates ranging from 0.3 to 4% [17–19, 32, 33]. After identification using multiplex PCR and verification by *rpoB* sequencing all isolates were classified as *A. butzleri*. This result is in agreement with previous studies in Turkey and Chile, where *A. butzleri* was the only species recovered from human feces [17, 32]. According to other authors, *A. cryaerophilus* and *A. skirrowii* can also be isolated from human stool samples [14, 15, 19]. However, the latter is only rarely detected due to slow growth on culture media and overgrowth by other bacteria, while the prevalence of *A. cryaerophilus* is up to 6.7-fold lower compared to *A. butzleri* [14, 17, 19, 33]. These are probably the main factors that caused lower species diversity in this study.

Improper hygienic practices at different stages of food supply chain may result in food contamination with *Arcobacter* spp. Handling and consumption of contaminated food products is considered as one of the main risk factors for human infection [7, 34]. The reported prevalence of *Arcobacter* spp. in foods varies greatly among different studies. However, most studies agree that the contamination rates of poultry meat are higher in comparison to red meat, raw cow milk and vegetables [23, 35, 36]. As reviewed by Hsu and Lee [9], *Arcobacter* spp. are more frequently found in food products of animal origin with the highest weighted mean prevalence in chicken meat (45.2%), followed by dairy products (36.4%), pork (36.3%), seafood (32.3%), beef (31.2%) and vegetables (14%). In this study, *Arcobacter* spp. were isolated from all tested food products (chicken meat, raw cow milk and RTE salad mixes), with an overall prevalence of 28.5% (152 of 534 samples). As expected, chicken meat showed the highest contamination levels (36%, 119/331), followed by raw milk (25%, 26/104) and RTE salads (7.1%, 7/99). Part of these results are in agreement with studies from Malaysia and Italy where *Arcobacter* was detected in 39% (48/123) of chicken meat and in 21.6% (8/37) of raw cow milk samples [37, 38]. According to other authors, the reported prevalence of *Arcobacter* spp. in chicken meat and raw cow milk ranged from 12 to 85.7% and from 4.1 to 46%, respectively [18, 39–41]. The isolation rate of *Arcobacter* in RTE salad mixes was lower in comparison with studies conducted in Italy and Portugal (i.e. ranging from 27.5 to 47.6%), but higher than the reported contamination of leafy green vegetables (4.4%, 4/90) from a study in South Korea [23, 42, 43]. Regarding the distribution of species based on sample type, *A. butzleri* was the only species detected in raw cow milk and the most commonly isolated species in chicken meat (114 out of 119 isolates), whereas in RTE packaged vegetables the most common was *A. cryaerophilus* (5 out of 7 isolates). *A. skirrowii* was not recovered from tested food samples. These results are in concordance with previous studies that reported *A. butzleri* as the predominant or the only species (75.4–100% of isolates) detected in chicken meat and raw cow milk. *A. cryaerophilus* was the second most commonly isolated species (0–21.5% of isolates), while *A. skirrowii* was rarely found (0–3.1% of isolates) [38–41]. The ability of *A. butzleri* to grow in low temperatures (4–10 ºC), attach to various pipe surfaces (i.e. stainless steel, copper and plastic), form biofilms and survive sanitizing procedures explains its persistence in the food processing environment and high isolation rates [44–46]. In case of RTE salads, the higher prevalence of *A. cryaerophilus* was not reported by previous studies. During our survey, pre-washed RTE salad mix samples were tested; therefore, higher *A. cryaerophilus* occurrence in vegetables might be associated with a higher capacity to adhere and survive on plant surfaces.

Contaminated water is considered as another important risk factor for public health, and it has been estimated that 63% of *A. butzleri* infections in humans are related to the consumption of or contact with contaminated water [6]. *Arcobacter* spp. were isolated from 36 out of 128 (28.1%) examined environmental water samples. This finding is consistent with a study from Canada, where *Arcobacter* was detected in 25.6% (173/676) of surface water samples [47]. However, the prevalence in environmental waters varies greatly across studies, with rates ranging from 20.8 to 58.6% [48, 49]. Out of 36 *Arcobacter* isolates, *A. butzleri* was the most prevalent species (n = 30) followed by *A. cryaerophilus* (n = 6), which is in accordance with other studies [49, 50].

Differences between reported *Arcobacter* prevalence rates in various sources may be due to numerous factors, such as examined sample sizes, geographic and seasonal variation, implemented hygiene protocols and sanitation procedures on farms and food processing facilities, patient populations, sensitivity and specificity of used detection methods. Due to the lack of standard isolation and cultural identification protocols, the latter aspect is of particular importance. According to previous studies, factors like including a pre-enrichment step, media composition and incubation conditions may cause differences in recovery rates ranging from 7.1 to 38% [43, 51–53]. Furthermore, it should be taken into consideration that only stool samples of inpatients were included in this study. *Arcobacter* infections are generally mild and do not require hospitalization, hence the overall prevalence might be higher than the one reported here. Nonetheless, *Arcobacter* was frequently isolated from chicken meat, environmental water, raw cow milk and RTE salads, which is consistent with previous reports.

### Antimicrobial susceptibility of isolated bacteria

At the European Union (EU) level, protocols that were developed by the European Food Safety Authority (EFSA) and the European Centre for Disease Prevention and Control (ECDC) are mainly focused on the harmonized monitoring of antimicrobial resistance in *Campylobacter* and *Salmonella* from various sources (i.e. food, food-producing animals and humans) [54]. In contrast to these zoonotic pathogens, the AST of *Arcobacter* is not standardized (i.e. there are no reference protocols or defined standard interpretive criteria). Therefore, data on antimicrobial susceptibility of *Arcobacter* spp. are scarce. Furthermore, the use of different testing methods and breakpoints hinder harmonized monitoring or comparative analysis and can result in therapeutic misguidance. Nevertheless, recent reports have indicated resistance of *Arcobacter* spp., isolated from food products, environment and human clinical samples, to several classes of antibiotics (i.e. macrolides, fluoroquinolones, lincosamides, tetracyclines and penicillins) [24, 55–57]. In these studies, resistance was determined by applying European Committee on Antimicrobial Susceptibility Testing (EUCAST) breakpoints for *Campylobacter*, *Enterobacteriaceae* and non-species related breakpoints, or Clinical and Laboratory Standards Institute (CLSI) breakpoints for *Campylobacter*, *Enterobacteriaceae* and *Staphylococcus* spp.

In our study, two different methods were used for the isolation of *Arcobacter* spp. from food products, environmental water and human stool samples. However, strains from different sources showed similar MIC distribution patterns (data not shown). Therefore, MIC data were aggregated and compared with EUCAST ECOFFs for *C. jejuni* [29]. Although the average nucleotide identity (ANI) between *C. jejuni* subsp. *jejuni* NCTC 11,168 and *A. butzleri* RM4018 is around 67% [58], *Campylobacter* is the most closely related genus to *Arcobacter* for which ECOFFs are available. Regardless of species, none of the tested *Arcobacter* isolates showed elevated MICs for gentamicin and tetracycline. These results are in concordance with previous studies from Belgium, Spain and Iran, where the determined resistance rates for gentamicin and tetracycline were between 0 and 3.6% and 0–11%, respectively [55, 56, 59]. In general, aminoglycosides (i.e. gentamicin, kanamycin and streptomycin) are highly effective against *Arcobacter* spp. and, therefore, are recommended for the treatment of severe infections [7]. However, in case of tetracycline, higher resistance rates (up to 90.5%) were recently reported [23].

Azithromycin is more effective than erythromycin against *Campylobacter*, which is reflected in 16-fold lower ECOFF value. Both of these antibiotics belong to the class of macrolides; thus, the changes (i.e. methylation or mutations) in ribosomal target sites and drug efflux usually cause cross-resistance in *Campylobacter* spp. [60]. Surprisingly, *Arcobacter* spp. AST revealed equal or up to 16 times higher azithromycin MIC values in comparison with those of erythromycin for 145 (69.7%; data not shown) isolates. Furthermore, MIC data for azithromycin were distributed bimodally, while an unimodal distribution for erythromycin was found. After applying *C. coli* EUCAST breakpoints, Van den Abeele et al. [56] found that 21.7% (23/106) of the *Arcobacter* strains were resistant to erythromycin. This finding is in agreement with our results, as 42 isolates (20.2%) had MICs > 8 µg/ml. According to other authors, from 2.8 to 100% of tested *Arcobacter* strains were resistant toward this antibiotic [59, 61]. High resistance rates pose a serious risk to public health as erythromycin is critically important for treatment of campylobacteriosis and it was suggested to be used in *Arcobacter* infections [62]. As described in previous studies, we also found that the majority of azithromycin MICs (96.2%) were equal to or above the *C. jejuni* ECOFF (0.25 µg/ml) [19, 56]. MIC data for azithromycin indicated the presence of a subpopulation with reduced susceptibility. Therefore, elevated MICs (≥ 8 µg/ml) were determined for 130 (67.7%) *A. butzleri* and 2 (12.5%) *A. cryaerophilus* strains. Similarly, Brückner et al. [19] observed elevated MIC values (> 8 µg/ml) for 54.2% *A. butzleri* and 10% *A. cryaerophilus* strains. Divergent MIC distribution patterns for macrolides are consistent with the results of a recent study from Germany that tested the *in vitro* susceptibility of clinical *Arcobacter* strains using the same methodology [19]. Additionally, erythromycin MIC values peaked at 4 µg/ml, while azithromycin MIC distribution was characterized by two peaks at 1 µg/ml and 16 µg/ml, which is also in agreement with our results. The authors have hypothesized that an amino acid substitution (A86E) in ribosomal protein L22 and the absence of mutations (A2074T or A2075G) in 23 S rRNA gene may result in resistance to azithromycin and susceptibility to erythromycin, which is seen in *Campylobacter* [19, 63]. AST of clinical *Legionella pneumophila* strains showed that there is a correlation between reduced susceptibility to azithromycin and the presence of the *lpeAB* genes encoding a macrolide efflux pump [64]. Although *Arcobacter* spp. do not possess these genes, the presence of *lpeAB* functional homologs (encoding MacAB-TolC) was already reported [58]. However, whole genome sequence-based analysis of *Arcobacter* is needed in order to determine the genetic mechanisms affecting the MICs of different macrolides.

According to EFSA and ECDC [54], ciprofloxacin resistance increased during the period from 2015 to 2019 in *C. jejuni* strains isolated from humans. In 2019, the reported resistance at EU level for *C. jejuni* and *C. coli* from various sources (i.e. humans, poultry, broiler meat) was between 61.5 and 90% and 61.2–89.4%, respectively. In comparison with *Campylobacter*, the resistance rates in *Arcobacter* are lower (ranging from 0 to 27.4%) [24, 56, 57]. Results of this study are in accordance with previous reports as only 18 (8.7%) *Arcobacter* isolates had elevated MICs (≥ 8 µg/ml), while the rest displayed low values that ranged between 0.032 and 1 µg/ml. The majority of strains (16/18) that had elevated MIC values were isolated from chicken meat. This result can be explained by the use of fluoroquinolones in poultry rearing [23]. A slightly higher percentage of *A. butzleri* isolates (8.9%) showed reduced susceptibility in comparison to *A. cryaerophilus* (6.3%), which is in line with a study by Rahimi et al. [59]. In case of ampicillin, high MICs (≥ 24 µg/ml) were determined for 46.9% of *A. butzleri* isolates, while only one *A. cryaerophilus* strain (6.3%) showed a MIC of 24 µg/ml. This result is in agreement with previous studies reporting high MICs for *A. butzleri* and *A. cryaerophilus* strains with rates of 42–100% and 0–23.3%, respectively [17, 19, 56]. Furthermore, a majority (23/30, 76.7%) of *A. butzleri* strains isolated from environmental water showed MICs that ranged from 24 µg/ml to > 256 µg/ml. High rates of resistance (94.4–100%) were observed in previous studies involving *A. butzleri* isolates from aquatic environment [24, 65].

According to our results, in case of ciprofloxacin, the *C. jejuni* ECOFF (0.5 µg/ml) could be applied for *Arcobacter* as isolates with MICs ranging from 0.032 to 0.5 µg/ml formed a wild-type subpopulation (i.e. bacteria without acquired resistance mechanisms). This result is in agreement with previous reports [19, 66]. However, for gentamicin, tetracycline, erythromycin, azithromycin and ampicillin, various rates of presumptive wild-type isolates (i.e. 35.6%, 70.2%, 39.6%, 89.5% and 66%, respectively; Fig. 1) had MICs that were above the ECOFF values for *C. jejuni*. Therefore, *Arcobacter* ECOFFs for these antimicrobials may be higher and should be reassessed.

### Prevalence of putative virulence genes

Although *A. butzleri* and *A. cryaerophilus* are considered as emerging zoonotic pathogens, data on virulence and pathogenic mechanisms is still limited [7]. The prevalence rates of putative virulence genes among *Arcobacter* spp. isolated from human, water and food samples were previously reported by several authors [25, 43, 67, 68]. However, this is the first study reporting the occurrence of virulence genes in *Arcobacter* strains isolated from different sources in Lithuania.

We examined *A. butzleri* and *A. cryaerophilus* isolates for the presence of ten genes (*mviN*, *cadF*, *cj1349*, *ciaB*, *pldA*, *hecA*, *hecB*, *tlyA*, *irgA* and *iroE*) that are homologous to virulence factors in *C. jejuni* and other pathogens. The *mviN* gene encodes a protein essential for peptidoglycan biosynthesis. Genes *cadF* and *cj1349* encode outer membrane proteins, which promote the binding of bacteria to intestinal epithelial cells, while *Campylobacter* invasive antigen B (CiaB) contributes to host cell invasion. The *hecA* encodes for an adhesin of the filamentous hemagglutinin family. Three genes, namely *pldA* (encoding the outer membrane phospholipase A), *hecB* (encoding hemolysin activation protein) and *tlyA* (encoding hemolysin), are associated with lysis of erythrocytes. Genes *irgA* and *iroE* encode functional components (iron-regulated outer membrane protein and periplasmic enzyme) of iron acquisition system and therefore are required for establishing and maintaining infections [28]. However, it is still unknown whether *Arcobacter* spp. putative virulence factors have functions similar to those of their homologues in other pathogens. Regardless of isolation source, six genes, namely *ciaB*, *mviN*, *pldA*, *tlyA*, *cj1349* and *cadF*, were identified in most or even all *A. butzleri* isolates (100, 97.9, 95.8, 99, 99 and 100%, respectively). The high occurrence of these genes (ranging between 77.5 and 100%) was reported in previous studies after testing *A. butzleri* isolates from human stool, food products and in-line milk filters of cow dairy farms [22, 25, 67, 69]. The remaining four genes, i.e., *hecA*, *hecB*, *irgA* and *iroE*, were less prevalent. Higher *cadF*, *ciaB*, *cj1349*, *mviN*, *pldA* and *tlyA* detection rates in comparison with *irgA*, *iroE*, *hecA*, and *hecB* are consistent between most of published studies [22, 67, 68, 70]. In general, the *irgA* gene showed the lowest occurrence rate (12.5%) and was not detected in isolates from human stool, raw cow milk, and RTE salad mixes. Similar prevalence rates (ranging from 7.1 to 17.6%) were reported previously [22, 55, 69]. The presence of *irgA* gene in *A. butzleri* from raw cow milk and RTE vegetables was rarely investigated; however, Girbau et al. [67] and Mottola et al. [42] did not detect *irgA* in strains that were isolated from these sources, which is in line with our study. The occurrence of *hecA* (20.3%), *hecB* (38.5%) and *iroE* (18.8%) genes is similar to that reported by other authors (ranging between 10.8 and 31.3, 29–38.8 and 12–30%, respectively) [22, 25, 69, 71]. Surprisingly, the presence of *hecA*, *irgA* and *iroE* was considerably lower in human stool and food isolates compared with environmental water isolates. This is in agreement with Karadas et al. [68] who determined higher detection rates for *irgA* (44%), *hecA* (44%) and *iroE* (67%) in isolates from water in comparison to isolates originating from humans, pork, chicken meat, and minced meat. However, in contrast to Karadas et al. [68], our results revealed that the gene encoding hemolysin activation protein (*hecB*) was significantly more prevalent in strains from water and human clinical samples compared with strains from food (P < 0.05). This difference might be associated with the lower number of isolates tested in previous study. Fourteen *A. butzleri* isolates (7.3%), obtained from chicken meat (n = 7; 6.1%) and environmental water (n = 7; 23.3%), were found to carry all ten putative virulence genes. Slightly different rates (ranging from 1.7 to 22.5%) were determined in studies from Spain and Germany [67, 72]. This disparity might be due to differences in the origin of tested isolates.

In accordance with other reports [25, 42, 48, 67], we observed fewer virulence genes (n = 5) among *A. cryaerophilus* strains in comparison to *A. butzleri*. For *A. cryaerophilus*, irrespective of origin, two genes (*ciaB* and *mviN*) were detected in all isolates, whereas *cj1349* and *hecA* were present in 12.5%, and *tlyA* in 25% of isolates. The predominance of *ciaB* and *mviN* in *A. cryaerophilus* was reported in previous studies involving isolates from poultry meat, water and other sources [25, 71]. For bacteria originating from vegetables, the data on virulence gene distribution is limited to one study, which showed partial agreement with our results. In particular, the study from Italy reported the presence of *cadF* and *mviN* in all *A. cryaerophilus* isolates, while other seven genes (i.e. *ciaB*, *cj1349*, *irgA*, *hecA*, *tlyA*, *hecB* and *pldA*) were not detected [42]. According to other authors, the occurrence of *cj1349*, *hecA* and *tlyA* in *A. cryaerophilus* varies greatly with rates ranging between 0 and 76.9%, 0–30%, and 0–31.8% respectively [42, 67, 73]. Furthermore, *cadF* (6.8–61.5%), *pldA* (16.9–61.5%) and *irgA* (2.6–15.9%) were also identified in *A. cryaerophilus* [43, 70, 73]; however, we did not detect these genes among tested isolates. The above-mentioned virulence profile differences within *A. cryaerophilus* species might be associated with higher genomic heterogeneity in primer target sequences [70].

## Conclusions

In conclusion, the data of this study provide first insight into the prevalence, antimicrobial susceptibility and putative virulence gene profiles of *Arcobacter* spp. from inpatients, foods of animal origin (chicken meat and raw cow milk), ready-to-eat (RTE) salad mixes and environmental water in Lithuania. High contamination rates of meat, milk, water and, to a lesser extent, RTE salad mixes, and the presence of multiple virulence genes in isolated *Arcobacter*, highlights their potential role in the epidemiology of *Arcobacter* infections. Moreover, according to our results, *Arcobacter* should be considered as an etiological factor for human gastroenteritis. Fluoroquinolones and aminoglycosides were found to be more effective against *A. butzleri*, and *A. cryaerophilus* in comparison with macrolides, tetracyclines, and aminopenicillins. Antimicrobial susceptibility testing also revealed different distribution patterns of minimal inhibitory concentration for macrolides (azithromycin and erythromycin). However, further *in vitro*, *in vivo* and *in silico* whole genome sequence-based studies are needed in order to (i) identify genetic mechanisms causing reduced susceptibility to antimicrobial agents, (ii) to determine the potential role of tested virulence factors in the pathogenesis of *Arcobacter* infection, and (iii) to clarify the epidemiological situation in other geographic regions.

## Methods

### Sample collection

In this study, a total of 1862 samples were collected in the city of Kaunas, Lithuania. As it is summarized in Table 1, human stool, chicken meat, raw cow milk and environmental water samples were collected during a 12-month survey, while RTE salad mixes were tested for 11 months. In total, 1200 human stool samples were collected by Kaunas Clinical Hospital Microbiology Laboratory for the detection of *Arcobacter* spp. Stool samples were collected prior to antimicrobial treatment from inpatients with symptoms of gastroenteritis. All participants were de-identified by pseudonymization. Therefore, patient data (including medical history) were not accessible. Chicken meat (n = 331, including drumsticks and wings), raw cow milk (n = 104) and RTE salad mixes (n = 99) were purchased from different randomly selected retail establishments. Sampling of surface waters (n = 128, including lake and river water) was performed along public beach sites using sterile 50 ml conical tubes. Food and water samples were transported to the laboratory and processed within 2 to 4 h of collection. Stool samples were transported from the clinical microbiology laboratory and tested for the presence of *Arcobacter* spp. within 7 days of collection.

### Isolation of *Arcobacter* spp

Depending on the sample type, two different approaches were used for the detection of *Arcobacter*. Isolation of *Arcobacter* spp. from human clinical samples was carried out using selective enrichment method described by van Driessche et al. [74]. Briefly, 1 g of feces per sample was transferred to sterile test tubes and diluted with 9 ml (1:10 dilution) of selective enrichment broth containing *Arcobacter* broth (Oxoid, Thermo Fisher Scientific, Basingstoke, United Kingdom) (24 g/l), 50 ml/l lysed horse blood (Oxoid, Thermo Fisher Scientific), amphotericin B (10 mg/l), cefoperazone (16 mg/l), novobiocin (32 mg/l), trimethoprim (64 mg/l) and 5-fluorouracil (100 mg/l) (all Sigma–Aldrich, Steinheim, Germany). Then, the samples were mixed using a vortex mixer and incubated for 72 h at 30 °C in a microaerobic atmosphere. Microaerobic conditions were produced using CampyGen gas packs (Oxoid, Thermo Fisher Scientific). After incubation, 50 µl of enrichment broth was streaked onto *Arcobacter* selective agar plates (same composition as described above, with the exception of lysed horse blood) and incubated for 48 h (30 °C, microaerobic conditions). Typical *Arcobacter* colonies (small, circular with entire margins, convex and whitish-gray) were subcultured onto Mueller-Hinton agar (Oxoid, Thermo Fisher Scientific) plates supplemented with 50 ml/l defibrinated sheep blood (MHB) (Oxoid, Thermo Fisher Scientific) and incubated for 48 h (30 °C, microaerobic conditions).

Isolation of *Arcobacter* spp. from food products and water was performed using membrane filtration method as previously described by Atabay et al. [75]. Prior to enrichment, water samples were centrifuged (3,500 x g for 10 min) and pellets were resuspended in 10 ml of *Arcobacter* broth (AB) with selective supplement containing cefoperazone (8 mg/l), amphotericin B (10 mg/l) and teicoplanin (4 mg/l) (CAT, Oxoid, Thermo Fisher Scientific). Each food sample (1 g or 1 ml) was added to AB/CAT at a ratio of 1:10. Subsequently, all samples were thoroughly mixed and incubated for 48 h at 30 °C under microaerobic conditions. Following incubation, 300 µl from each enriched sample was transferred onto a 0.45 μm pore size mixed cellulose ester membrane filter (Frisenette, Knebel, Denmark) placed on the surface of MHB agar. After 1 h of passive filtration (30 °C, aerobic conditions), the filters were aseptically removed and plates were incubated at 30 °C in a microaerobic atmosphere. The plates were checked every 24 h (up to 7 days) for the presence of typical *Arcobacter* colonies. From each plate, five suspected colonies were subcultured onto MHB plates for 48 h at 30 °C in microaerobic conditions.

### Molecular identification and verification of *Arcobacter* isolates

Template DNA of presumptive *Arcobacter* isolates was prepared using PrepMan® Ultra Reagent (Applied Biosystems, Woolston, Warrington, United Kingdom) according to the manufacturer‘s specifications. Isolates were identified at species level using multiplex polymerase chain reaction (mPCR) previously described by Houf et al. [76]. Primers targeting 23 S and 16 S rRNA genes for the simultaneous identification of *A. cryaerophilus*, *A. butzleri* and *A. skirrowii* were used (Table 5). Amplification reaction mixture contained 2 µl template DNA, 12.5 µl of DreamTaq™ Green PCR Master Mix (2x) (Thermo Fisher Scientific, Vilnius, Lithuania), 1 µM of each primer ARCO R, BUTZ F, CRY 1, CRY 2, 0.5 µM of primer SKIR F and 8.25 µl of molecular grade water (Thermo Fisher Scientific) in a total reaction volume of 25 µl. Prior to cycling, samples underwent initial denaturation step at 94 °C for 2 min. This step was followed by 32 PCR cycles, consisting of denaturation at 94 °C for 45 s, annealing at 61 °C for 45 s and extension at 72 °C for 30 s and a final elongation step at 72 °C for 5 min. DNA of *A. butzleri* (ATCC 49,616), *A. cryaerophilus* (ATCC 43,158) and *A. skirrowii* (ATCC 51,132) were used as positive control, while molecular grade water (Thermo Fisher Scientific) was used as negative control. Separation of amplification products was performed using horizontal electrophoresis in 2% agarose in 1xTris-Borate-EDTA (TBE) buffer. The gels were stained with ethidium bromide and visualized under UV light.

**Table 5:** List of primers used in this study

Target gene	Primer pair	Primer sequence (5’–3’)	Amplicon size (bp)	References
Species identification				
* A. butzleri*	BUTZ F	CCTGGACTTGACATAGTAAGAATGA	401	Houf et al. [76]
16 S rRNA	ARCO R	CGTATTCACCGTAGCATAGC		
* A. skirrowii*	SKIR F	GGCGATTTACTGGAACACA	641	
16 S rRNA	ARCO R	CGTATTCACCGTAGCATAGC		
* A. cryaerophilus*	CRY1	TGCTGGAGCGGATAGAAGTA	257	
23 S rRNA	CRY2	AACAACCTACGTCCTTCGAC		
Verification				
* rpoB*	CamrpoB-L	CCAATTTATGGATCAAAC	524	Korczak et al. [77]
	RpoB-R	GTTGCATGTTNGNACCCAT		
Detection of putative virulence genes				
* mviN*	mviN-F	TGCACTTGTTGCAAAACGGTG	294	Whiteduck-Leveillee et al. [78]
	mviN-R	TGCTGATGGAGCTTTTACGCAAGC		
* cadF*	cadF-F	TTACTCCTACACCGTAGT	283	Douidah et al. [70]
	cadF-R	AAACTATGCTAACGCTGGTT		
* cj1349*	cj1349-F	CCAGAAATCACTGGCTTTTGAG	659	Whiteduck-Leveillee et al. [78]
	cj1349-R	GGGCATAAGTTAGATGAGGTTCC		
* ciaB*	ciaB-F	TGGGCAGATGTGGATAGAGCTTGGA	284	
	ciaB-R	TAGTGCTGGTCGTCCCACATAAAG		
* pldA*	pldA-F	TTGACGAGACAATAAGTGCAGC	293	
	pldA-R	CGTCTTTATCTTTGCTTTCAGGGA		
* hecA*	hecA-F	GTGGAAGTACAACGATAGCAGGCTC	537	
	hecA-R	GTCTGTTTTAGTTGCTCTGCACTC		
* hecB*	hecB-F	CTAAACTCTACAAATCGTGC	528	
	hecB-R	CTTTTGAGTGTTGACCTC		
* tlyA*	tlyA-F	CAAAGTCGAAACAAAGCGACTG	230	
	tlyA-R	TCCACCAGTGCTACTTCCTATA		
* irgA*	irgA-F	TGCAGAGGATACTTGGAGCGTAACT	437	
	irgA-R	GTATAACCCCATTGATGAGGAGCA		
* iroE*	iroE-F	AATGGCTATGATGTTGTTTAC	415	Karadas et al. [68]
	iroE-R	TTGCTGCTATGAAGTTTTG		

Verification of identified isolates was ensured by *rpoB* gene sequencing previously described by Korczak et al. [77]. Briefly, *rpoB* gene amplification was performed in a 50 µl PCR-mixture containing 4 µl of template DNA, 1x PCR buffer, 0.75 U of Taq polymerase, 0.2 mM of each deoxynucleoside triphosphate (dNTP), 2.5 mM of MgCl_2_ (all from Thermo Fisher Scientific), 0.4 µM of each primer CamrpoB-L and RpoB-R. Before cycling, samples were subjected to initial denaturation step at 95 °C for 3 min. PCR involved 35 cycles with following conditions: denaturation at 94 °C for 30 s, annealing at 54 °C for 30 s and extension at 72 °C for 30 s. Last cycle was followed by a final elongation step at 72 °C for 5 min. Amplified products were separated and visualized as described above. Purification of PCR products was performed using GeneJET PCR Purification Kit (Thermo Fisher Scientific) according to manufacturer’s specifications. Around 30 ng purified PCR products were sequenced by GATC (Eurofins GATC Biotech, Konstanz, Germany). Identification of species was performed by comparing query *rpoB* sequences with BLAST database (NCBI).

### Antimicrobial susceptibility testing

All identified and verified isolates were tested for susceptibility to six antimicrobial agents (azithromycin, ampicillin, ciprofloxacin, gentamicin, erythromycin and tetracycline) by gradient strip diffusion method (E-test^TM^, bioMérieux, Nürtingen, Germany). The AST assays were performed according to the manufacturer’s instructions with minor modifications. Briefly, *Arcobacter* isolates were grown on MHB agar plates under microaerobic atmosphere for 48 h at 30 °C. A small amount of colony material from every plate was transferred to tubes with 2 ml of Brucella broth (BB) (Biolife, Milan, Italy) and incubated overnight (30 °C) under microaerobic conditions. These precultures were used to achieve an inoculum of approximately 1 × 10^8^ colony forming units (CFU) per ml. Because of the slow growth of *A. cryaerophilus* isolates, three overnight cultures per isolate were prepared. After overnight incubation all cultures from one isolate were pooled (6 ml), centrifuged (16,000 x g for 5 min) and the pellets were resuspended in 0.6 ml of BB to yield analogous inoculum concentrations. *Escherichia coli* ATCC 25,922 was used as a quality control in every test run (cultured on MHB for 48 h and precultured overnight in BB for 24 h at 37 °C in an aerobic atmosphere). Test strips were applied to MHB agar plates after inoculating them with 100 µl of overnight culture. Minimum inhibitory concentrations (MICs) were determined after 48 h incubation at 30 °C under microaerobic conditions (37 °C and aerobic atmosphere for the reference strain of *E. coli*). Only agar plates with a confluent bacterial lawn were evaluated.

### Detection of virulence genes

The presence of ten putative *Arcobacter* virulence genes was determined by PCR. All primers used are listed in Table 4. PCR protocols for partial amplification of *cj1349*, *ciaB*, *mviN*, *pldA*, *tlyA*, *irgA*, *hecA* and *hecB* were used as previously described by Whiteduck-Leveillee et al. [78]. Briefly, PCR assay was carried out in 25 µl volume reaction mixture containing 2 µl template DNA, 12.5 µl of DreamTaq™ Green PCR Master Mix (2x) and 0.1 µM of each forward and reverse primer. PCR conditions were as follows: initial denaturation (95 °C for 4 min), 30 cycles of amplification (denaturation at 95 °C for 30 s, annealing at 56 °C for 45 s and extension at 72 °C for 45 s) and final elongation (72 °C for 5 min). Partial amplification of *cadF* and *iroE* was carried out using the protocol described by Karadas et al. [68]. The reaction mixture was of the same composition as described above, except that primers were used at 1 µM. The reaction involved initial denaturation (95 °C for 4 min), followed by 30 cycles of amplification (95 °C for 30 s, 50 °C for 30 s and 72 °C for 30 s) and ended up with a final elongation step (72 °C for 5 min). Amplification products were separated by horizontal electrophoresis in 2% agarose in 1xTBE buffer. The gels were stained with ethidium bromide. The presence of fragments was checked under a UV trans-illuminator.

### Statistical analysis

Data were analyzed by using Microsoft Office Excel 2016 (Microsoft Corp., Redmond, WA, US) and IBM SPSS Statistics 26.0 software package (IBM Corp., Armonk, NY, US). The Pearson’s chi-squared test and Fisher’s exact test were performed in order to compare the differences between prevalence rates, and to analyze the association of the ten putative virulence genes in *Arcobacter* isolates with their biological origin. In both cases, statistical hypotheses were tested between two sources (in various combinations) and differences were considered significant if P < 0.05.

## Data Availability

All data generated or analyzed during this study are included in this published article.

## References

[1] Vandamme P, Falsen E, Rossau R, Hoste B, Segers P, Tytgat R (1991). Revision of *Campylobacter*, *Helicobacter*, and *Wolinella* taxonomy: emendation of generic descriptions and proposal of *Arcobacter* gen. nov. Int J Syst Bacteriol.

[2] Ferreira S, Oleastro M, Domingues F (2019). Current insights on *Arcobacter butzleri* in food chain. Curr Opin Food Sci.

[3] Pérez-Cataluña A, Salas-Massó N, Diéguez AL, Balboa S, Lema A, Romalde JL (2018). Revisiting the taxonomy of the genus *Arcobacter*: Getting order from the chaos. Front Microbiol.

[4] On SLW, Miller WG, Biggs PJ, Cornelius AJ, Vandamme P (2020). A critical rebuttal of the proposed division of the genus *Arcobacter* into six genera using comparative genomic, phylogenetic, and phenotypic criteria. Syst Appl Microbiol.

[5] Kerkhof PJ, Van den Abeele AM, Strubbe B, Vogelaers D, Vandamme P, Houf K (2021). Diagnostic approach for detection and identification of emerging enteric pathogens revisited: the *(Ali)arcobacter lanthieri* case. New Microbes New Infect.

[6] Ramees TP, Dhama K, Karthik K, Rathore RS, Kumar A, Saminathan M (2017). *Arcobacter*: an emerging food-borne zoonotic pathogen, its public health concerns and advances in diagnosis and control—a comprehensive review. Vet Q.

[7] Chieffi D, Fanelli F, Fusco V (2020). *Arcobacter butzleri*: Up-to‐date taxonomy, ecology, and pathogenicity of an emerging pathogen. Compr Rev Food Sci Food Saf.

[8] Collado L, Figueras MJ (2011). Taxonomy, epidemiology, and clinical relevance of the genus *Arcobacter*. Clin Microbiol Rev.

[9] Hsu TTD, Lee J (2015). Global distribution and prevalence of *Arcobacter* in food and water. Zoonoses Public Health.

[10] Niedermeyer JA, Miller WG, Yee E, Harris A, Emanuel RE, Jass T (2020). Search for *Campylobacter* spp. reveals high prevalence and pronounced genetic diversity of *Arcobacter butzleri* in floodwater samples associated with Hurricane Florence in North Carolina, USA. Appl Environ Microbiol.

[11] Ferreira S, Queiroz JA, Oleastro M, Domingues FC. Insights in the pathogenesis and resistance of *Arcobacter*: a review. Crit Rev Microbiol. 2015;1–20. 10.3109/1040841X.2014.954523 25806423

[12] Arguello E, Otto CC, Mead P, Babady NE (2015). Bacteremia caused by *Arcobacter butzleri* in an immunocompromised host. J Clin Microbiol.

[13] Yap DYH, Kwan LPY, To KKW, Chan TM (2013). *Arcobacter* peritonitis after fluoroscopic repositioning of a Tenckhoff catheter. Perit Dial Int J Int Soc Perit Dial.

[14] Van den Abeele AM, Vogelaers D, Van Hende J, Houf K (2014). Prevalence of *Arcobacter* species among humans, Belgium, 2008–2013. Emerg Infect Dis.

[15] Wybo I, Breynaert J, Lauwers S, Lindenburg F, Houf K (2004). Isolation of *Arcobacter skirrowii* from a patient with chronic diarrhea. J Clin Microbiol.

[16] Samie A, Obi CL, Barrett LJ, Powell SM, Guerrant RL (2007). Prevalence of *Campylobacter* species, *Helicobacter pylori* and *Arcobacter* species in stool samples from the Venda region, Limpopo, South Africa: studies using molecular diagnostic methods. J Infect.

[17] Kayman T, Abay S, Hizlisoy H, Atabay Hİ, Diker KS, Aydin F (2012). Emerging pathogen *Arcobacter* spp. in acute gastroenteritis: molecular identification, antibiotic susceptibilities and genotyping of the isolated arcobacters. J Med Microbiol.

[18] Patyal A, Rathore RS, Mohan HV, Dhama K, Kumar A (2011). Prevalence of *Arcobacter* spp. in humans, animals and foods of animal origin including sea food from India. Transbound Emerg Dis.

[19] Brückner V, Fiebiger U, Ignatius R, Friesen J, Eisenblätter M, Höck M (2020). Prevalence and antimicrobial susceptibility of *Arcobacter* species in human stool samples derived from out- and inpatients: the prospective German *Arcobacter* prevalence study Arcopath. Gut Pathog.

[20] Figueras MJ, Levican A, Pujol I, Ballester F, Rabada Quilez MJ, Gomez-Bertomeu F (2014). A severe case of persistent diarrhoea associated with *Arcobacter cryaerophilus* but attributed to *Campylobacter* sp. and a review of the clinical incidence of *Arcobacter* spp. New Microbes New Infect.

[21] Ferreira S, Luís Â, Oleastro M, Pereira L, Domingues FC (2019). A meta-analytic perspective on *Arcobacter* spp. antibiotic resistance. J Glob Antimicrob Resist.

[22] Rathlavath S, Kohli V, Singh AS, Lekshmi M, Tripathi G, Kumar S (2017). Virulence genotypes and antimicrobial susceptibility patterns of *Arcobacter butzleri* isolated from seafood and its environment. Int J Food Microbiol.

[23] Vicente-Martins S, Oleastro M, Domingues FC, Ferreira S (2018). *Arcobacter* spp. at retail food from Portugal: Prevalence, genotyping and antibiotics resistance. Food Control.

[24] Šilha D, Pejchalová M, Šilhová L (2017). Susceptibility to 18 drugs and multidrug resistance of *Arcobacter* isolates from different sources within the Czech Republic. J Glob Antimicrob Resist.

[25] Brückner V, Fiebiger U, Ignatius R, Friesen J, Eisenblätter M, Höck M (2020). Characterization of *Arcobacter* strains isolated from human stool samples: results from the prospective German prevalence study Arcopath. Gut Pathog.

[26] Ho HTK, Lipman LJA, Hendriks HGCJM, Tooten PCJ, Ultee T, Gaastra W (2007). Interaction of *Arcobacter* spp. with human and porcine intestinal epithelial cells. FEMS Immunol Med Microbiol.

[27] Bücker R, Troeger H, Kleer J, Fromm M, Schulzke J (2009). *Arcobacter butzleri* induces barrier dysfunction in intestinal HT-29/B6 cells. J Infect Dis.

[28] Miller WG, Parker CT, Rubenfield M, Mendz GL, Wösten MMSM, Ussery DW (2007). The complete genome sequence and analysis of the Epsilonproteobacterium *Arcobacter butzleri*. PLoS ONE.

[29] European Committee on Antimicrobial Susceptibility Testing. Data from the EUCAST MIC distribution website. 2021. https://www.eucast.org. Accessed 10 Mar 2021.

[30] International Commission on Microbiological Specifications for Foods (ICMSF), Tompkin RB, editors. Microorganisms in foods 7: microbiological testing in food safety management. New York: Kluwer Academic/Plenum Publishers; 2002. p. 171.

[31] Ferreira S, Júlio C, Queiroz JA, Domingues FC, Oleastro M (2014). Molecular diagnosis of *Arcobacter* and *Campylobacter* in diarrhoeal samples among Portuguese patients. Diagn Microbiol Infect Dis.

[32] Fernandez H, Villanueva MP, Mansilla I, Gonzalez M, Latif F (2015). *Arcobacter butzleri* and *A. cryaerophilus* in human, animals and food sources, in southern Chile. Braz J Microbiol.

[33] Vandenberg O, Dediste A, Houf K, Ibekwem S, Souayah H, Cadranel S (2004). *Arcobacter* species in humans. Emerg Infect Dis.

[34] Shange N, Gouws P, Hoffman LC (2019). *Campylobacter* and *Arcobacter* species in food-producing animals: prevalence at primary production and during slaughter. World J Microbiol Biotechnol.

[35] Nieva-Echevarria B, Martinez-Malaxetxebarria I, Girbau C, Alonso R, Fernández-Astorga A (2013). Prevalence and genetic diversity of *Arcobacter* in food products in the north of Spain. J Food Prot.

[36] Shah AH, Saleha AA, Zunita Z, Murugaiyah M (2011). *Arcobacter*—an emerging threat to animals and animal origin food products?. Trends Food Sci Technol..

[37] Amare LB, Saleha AA, Zunita Z, Jalila A, Hassan L (2011). Prevalence of *Arcobacter* spp. on chicken meat at retail markets and in farm chickens in Selangor, Malaysia. Food Control.

[38] Traversa A, Gallina S, Martucci F, Boteva C, Baioni E, Maurella C (2019). *Arcobacter* spp. in raw milk from vending machines in Piedmont and occurrence of virulence genes in isolates. Ital J Food Saf.

[39] Zacharow I, Bystroń J, Wałecka-Zacharska E, Podkowik M, Bania J (2015). Prevalence and antimicrobial resistance of *Arcobacter butzleri* and *Arcobacter cryaerophilus* isolates from retail meat in Lower Silesia region, Poland. Pol J Vet Sci.

[40] Marta C, Giovanni N, Angela M, Loredana C, Elisabetta B, Laura D (2020). Large genetic diversity of *Arcobacter butzleri* isolated from raw milk in Southern Italy. Food Microbiol.

[41] Scullion R, Harrington CS, Madden RH (2006). Prevalence of *Arcobacter* spp. in raw milk and retail raw meats in Northern Ireland. J Food Prot.

[42] Mottola A, Bonerba E, Bozzo G, Marchetti P, Celano GV, Colao V (2016). Occurrence of emerging food-borne pathogenic *Arcobacter* spp. isolated from pre-cut (ready-to-eat) vegetables. Int J Food Microbiol..

[43] Kim NH, Park SM, Kim HW, Cho TJ, Kim SH, Choi C (2019). Prevalence of pathogenic *Arcobacter* species in South Korea: Comparison of two protocols for isolating the bacteria from foods and examination of nine putative virulence genes. Food Microbiol.

[44] Assanta MA, Roy D, Lemay M-J, Montpetit D (2002). Attachment of *Arcobacter butzleri*, a new waterborne pathogen, to water distribution pipe surfaces. J Food Prot.

[45] Kjeldgaard J, Jørgensen K, Ingmer H (2009). Growth and survival at chiller temperatures of *Arcobacter butzleri*. Int J Food Microbiol.

[46] Rasmussen L, Kjeldgaard J, Christensen J, Ingmer H (2013). Multilocus sequence typing and biocide tolerance of *Arcobacter butzleri* from Danish broiler carcasses. BMC Res Notes.

[47] Webb AL, Taboada EN, Selinger LB, Boras VF, Inglis GD (2017). Prevalence and diversity of waterborne *Arcobacter butzleri* in southwestern Alberta, Canada. Can J Microbiol.

[48] Laishram M, Rathlavath S, Lekshmi M, Kumar S, Nayak BB (2016). Isolation and characterization of *Arcobacter* spp. from fresh seafood and the aquatic environment. Int J Food Microbiol..

[49] Collado L, Inza I, Guarro J, Figueras MJ (2008). Presence of *Arcobacter* spp. in environmental waters correlates with high levels of fecal pollution. Environ Microbiol.

[50] Collado L, Kasimir G, Perez U, Bosch A, Pinto R, Saucedo G (2010). Occurrence and diversity of *Arcobacter* spp. along the Llobregat River catchment, at sewage effluents and in a drinking water treatment plant. Water Res.

[51] Rathlavath S, Kumar S, Nayak BB (2017). Comparative isolation and genetic diversity of *Arcobacter* sp. from fish and the coastal environment. Lett Appl Microbiol.

[52] Fallas-Padilla KL, Rodríguez-Rodríguez CE, Jaramillo HF, Echandi MLA (2014). *Arcobacter*: comparison of isolation methods, diversity, and potential pathogenic factors in commercially retailed chicken breast meat from Costa Rica. J Food Prot.

[53] Scullion R, Harrington CS, Madden RH (2004). A comparison of three methods for the isolation of *Arcobacter* spp. from retail raw poultry in Northern Ireland. J Food Prot.

[54] European Food Safety Authority, European Centre for Disease Prevention and Control (2021). The European Union Summary Report on Antimicrobial Resistance in zoonotic and indicator bacteria from humans, animals and food in 2018/2019. EFSA J.

[55] Pérez-Cataluña A, Tapiol J, Benavent C, Sarvisé C, Gómez F, Martínez B (2017). Antimicrobial susceptibility, virulence potential and sequence types associated with *Arcobacter* strains recovered from human faeces. J Med Microbiol.

[56] Van den Abeele AM, Vogelaers D, Vanlaere E, Houf K (2016). Antimicrobial susceptibility testing of *Arcobacter butzleri* and *Arcobacter cryaerophilus* strains isolated from Belgian patients. J Antimicrob Chemother.

[57] Fanelli F, Chieffi D, Di Pinto A, Mottola A, Baruzzi F, Fusco V (2020). Phenotype and genomic background of *Arcobacter butzleri* strains and taxogenomic assessment of the species. Food Microbiol.

[58] Müller E, Abdel-Glil MY, Hotzel H, Hänel I, Tomaso H (2020). *Aliarcobacter butzleri* from water poultry: insights into antimicrobial resistance, virulence and heavy metal resistance. Genes.

[59] Rahimi E (2014). Prevalence and antimicrobial resistance of *Arcobacter* species isolated from poultry meat in Iran. Br Poult Sci.

[60] Wei B, Kang M (2018). Molecular basis of macrolide resistance in *Campylobacter* strains isolated from poultry in South Korea. BioMed Res Int.

[61] Elmali M, Can HY (2017). Occurence and antimicrobial resistance of *Arcobacter* species in food and slaughterhouse samples. Food Sci Technol.

[62] Fanelli F, Di Pinto A, Mottola A, Mule G, Chieffi D, Baruzzi F (2019). Genomic characterization of *Arcobacter butzleri* isolated from shellfish: Novel insight into antibiotic resistance and virulence determinants. Front Microbiol.

[63] Zhao S, Tyson GH, Chen Y, Li C, Mukherjee S, Young S (2016). Whole-genome sequencing analysis accurately predicts antimicrobial resistance phenotypes in *Campylobacter* spp. Appl Environ Microbiol.

[64] Vandewalle-Capo M, Massip C, Descours G, Charavit J, Chastang J, Billy PA (2017). Minimum inhibitory concentration (MIC) distribution among wild-type strains of *Legionella pneumophila* identifies a subpopulation with reduced susceptibility to macrolides owing to efflux pump genes. Int J Antimicrob Agents.

[65] Sciortino S, Arculeo P, Alio V, Cardamone C, Nicastro L, Arculeo M (2021). Occurrence and antimicrobial resistance of *Arcobacter* spp. recovered from aquatic environments. Antibiotics.

[66] Riesenberg A, Frömke C, Stingl K, Feßler AT, Gölz G, Glocker EO (2017). Antimicrobial susceptibility testing of *Arcobacter butzleri*: development and application of a new protocol for broth microdilution. J Antimicrob Chemother.

[67] Girbau C, Guerra C, Martínez-Malaxetxebarria I, Alonso R, Fernández-Astorga A (2015). Prevalence of ten putative virulence genes in the emerging foodborne pathogen *Arcobacter* isolated from food products. Food Microbiol.

[68] Karadas G, Sharbati S, Hänel I, Messelhäußer U, Glocker E, Alter T (2013). Presence of virulence genes, adhesion and invasion of *Arcobacter butzleri*. J Appl Microbiol.

[69] Piva S, Gariano GR, Bonilauri P, Giacometti F, Decastelli L, Florio D (2017). Occurrence of putative virulence genes on *Arcobacter butzleri* isolated from three different environmental sites throughout the dairy chain. J Appl Microbiol.

[70] Douidah L, de Zutter L, Bare J, De Vos P, Vandamme P, Vandenberg O (2012). Occurrence of putative virulence genes in *Arcobacter* species isolated from humans and animals. J Clin Microbiol.

[71] Šilha D, Vacková B, Šilhová L (2019). Occurrence of virulence-associated genes in *Arcobacter butzleri* and *Arcobacter cryaerophilus* isolates from foodstuff, water, and clinical samples within the Czech Republic. Folia Microbiol (Praha).

[72] Lehmann D, Alter T, Lehmann L, Uherkova S, Seidler T, Gölz G (2015). Prevalence, virulence gene distribution and genetic diversity of *Arcobacter* in food samples in Germany. Berl Munch Tierarztl Wochenschr.

[73] Sekhar MS, Tumati SR, Chinnam BK, Kothapalli VS, Sharif NM (2017). Virulence gene profiles of *Arcobacter* species isolated from animals, foods of animal origin, and humans in Andhra Pradesh, India. Vet World.

[74] van Driessche E, Houf K, Hoof J, Zutter L, Vandamme P (2003). Isolation of *Arcobacter* species from animal feces. FEMS Microbiol Lett.

[75] Atabay HI, Aydin F, Houf K, Sahin M, Vandamme P (2003). The prevalence of *Arcobacter* spp. on chicken carcasses sold in retail markets in Turkey, and identification of the isolates using SDS-PAGE. Int J Food Microbiol.

[76] Houf K, Tutenel A, Zutter L, Hoof J, Vandamme P (2000). Development of a multiplex PCR assay for the simultaneous detection and identification of *Arcobacter butzleri*, *Arcobacter cryaerophilus* and *Arcobacter skirrowii*. FEMS Microbiol Lett..

[77] Korczak BM, Stieber R, Emler S, Burnens AP, Frey J, Kuhnert P (2006). Genetic relatedness within the genus *Campylobacter* inferred from *rpoB* sequences. Int J Syst Evol Microbiol.

[78] Whiteduck-Léveillée J, Cloutier M, Topp E, Lapen DR, Talbot G, Villemur R (2016). Development and evaluation of multiplex PCR assays for rapid detection of virulence-associated genes in *Arcobacter* species. J Microbiol Methods.

